# Frontline healthcare workers’ experiences of providing care during the COVID-19 pandemic at a COVID-19 centre in Bulawayo, Zimbabwe: A phenomenological study

**DOI:** 10.4102/curationis.v45i1.2292

**Published:** 2022-06-30

**Authors:** Idah Moyo, Azwihangwisi H. Mavhandu-Mudzusi, Clara Haruzivishe

**Affiliations:** 1 Department of HIV Services, Population Solutions for Health, Harare, Zimbabwe; 2 Department of Health Science, College of Human Studies, University of South Africa, Pretoria, South Africa; 3 Department of Graduate Studies and Research, College of Human Sciences, University of South Africa, Pretoria, South Africa; 4 College of Health Sciences, Faculty of Medicine and Health Sciences, University of Zimbabwe, Harare, Zimbabwe

**Keywords:** COVID-19, healthcare workers, experiences, phenomenology, providers of care

## Abstract

**Background:**

The coronavirus disease 2019 (COVID-19) pandemic has had a far-reaching, negative impact on healthcare systems worldwide. Healthcare workers play a critical role in the country’s healthcare delivery system, as they facilitate a continuum of care and containment of diseases such as the COVID-19 pandemic.

**Objectives:**

The aim of this study was to explore and describe the experiences of healthcare workers who provided care to COVID-19 patients at a central hospital in Zimbabwe.

**Method:**

The researchers used an interpretative phenomenological analysis design. In-depth interviews were conducted virtually with 10 frontline healthcare workers working at a COVID-19 centre in Zimbabwe. Data collection was guided by an interview guide. All audio-recorded interview data were transcribed verbatim into written text. Data analysis was conducted using an interpretative phenomenological analysis framework. An expert in qualitative research acted as an independent co-coder and conducted the open coding of each transcript.

**Results:**

Findings reveal inadequate preparation and training of healthcare providers before the commencement of duty, resources-related challenges and a lack of support as significant experiences of healthcare providers. Moreover, healthcare providers have been subjected to stigma and discrimination attached to COVID-19, resulting in psychological effects on frontline healthcare providers.

**Conclusion:**

The COVID-19 pandemic brings unique and challenging experiences for frontline healthcare workers, resulting in a physically and emotionally drained workforce. This study calls for comprehensive support in the form of counselling, reasonable work schedules, training and adequate provision of personal protective equipment.

## Introduction

Although the coronavirus disease 2019 (COVID-19) pandemic started as a localised outbreak in Wuhan, China, at the end of December 2019, the virus spread worldwide by March 2020 (World Health Organization [WHO] 2020a). On 11 March 2020, the WHO declared COVID-19 a global pandemic as a result of the deep concern regarding the alarming levels of its spread and severity and the slow global response to it (Hageman 2020). The pandemic had a severe impact on developed countries and poses a severe threat to African countries. This is because many countries in Africa have poor healthcare delivery systems (Dzinamarira, Dzobo & Chitungo 2020; The World Economic Forum 2020).

A global view of the COVID-19 pandemic shows the huge impact it has had on frontline healthcare workers. Studies have demonstrated that during pandemics, such as COVID-19, healthcare professionals that occupy the frontlines are at increased risk concerning their physical and mental health (Cullen et al. 2020; Shanafelt, Ripp & Trockel 2020). This is because of the excessive workload, long working hours, unpreparedness and the psychological strain emanating from the fear of contracting the virus or concerns about how their exposure will impact their families (Shanafelt et al. 2020).

According to the WHO (2020b), on 23 July 2020, about 10% of all COVID-19 cases, globally, were amongst healthcare workers. The same report indicated that, in the 40 African countries which had reported on COVID-19 infections, more than 10 000 healthcare workers had contracted the COVID-19 virus. This highlights the importance of protecting health workers.

The burden of COVID-19 disease differs from country to country, with some countries more affected than others. The Ministry of Health and Child Care (2021) reported that, as of 14 June 2021, Zimbabwe had a cumulative number of 101 711 confirmed COVID-19 cases, 70 496 recoveries and 3280 fatalities. The country had a recovery rate of 69% and a case fatality ratio of 3.2%. The greatest burden of COVID-19 cases is in the country’s two biggest provinces, Harare and Bulawayo (Ministry of Health and Child Care 2021). This is attributed to the fact that they are the biggest in terms of geographic and population size. However, evidence has demonstrated that there is under-reporting of COVID-19 cases globally (Chen et al. 2021; Krantz & Rao 2020). This has been attributed to the fact that some COVID-19 cases may not be identified because of having mild symptoms and/or being asymptomatic (Alene et al. 2021; Paleker et al. 2021).

Healthcare workers play a critical role in the country’s healthcare delivery system, as they facilitate a continuum of care as well as containment of such pandemics as COVID-19 (Chang et al. 2020; Rusakaniko et al. 2021).

Previous studies on viral infections, such as the outbreaks of the Ebola virus disease, severe acute respiratory syndrome (SARS) and the Middle East respiratory syndrome, have demonstrated that these epidemics can have a negative impact on frontline healthcare workers. The studies found that these healthcare workers experienced stigma and discrimination, increased risks of infection, increased workloads and psychological distress (Brooks et al. 2018; Kim 2018; Raven, Wurie & Witter 2018).

Attempts have been made to explore and document the experiences of healthcare providers involved in COVID-19 management in other countries, such as the research by Liu et al. (2020) and Sun et al. (2020) in China, Okediran et al. (2020) in Nigeria and Mostafa, Sabry and Mostafa (2020) in Egypt. Whilst these studies provide important insights into the COVID-19 pandemic, they may not be reflective of the Zimbabwean context because the healthcare systems in these respective countries are different. What was different was that in all these studies in different countries, efforts were made to provide different forms of support such as online mental health support and/or strengthening the support system for healthcare workers providing care to COVID-19 patients. The provision of psychosocial support to healthcare workers was found to be a critical component during this and other pandemics (Ng et al. 2020; Raven et al. 2018). In the context of Zimbabwe, where the study was undertaken, very few studies have been undertaken (Mackworth-Young et al. 2021; Rusakaniko et al. 2021). This study explored and described the experiences of healthcare workers who provided care to COVID-19 patients at a central hospital in Zimbabwe.

## Methodology

### Design

Interpretative phenomenological analysis (IPA) design was employed to gain insight into the lived experiences of healthcare workers (nurses and doctors) in providing patient care during the COVID-19 pandemic. Through IPA, researchers have the best opportunity to understand the innermost deliberation of study participants’ ‘lived experiences’ (Alase 2017). The approach enabled study participants to narrate their stories based on their lived experiences (Creswell & Creswell 2018; Smith & Osborn 2015) and facilitated the adoption of an insider perspective. In addition, the researchers were able to explore and ask critical questions on certain aspects that are mentioned by the study participants to gain an in-depth understanding of their experiences (Smith & Osborn 2015).

### Study setting and participants

The study setting was a central hospital in Bulawayo, Zimbabwe. The hospital has a COVID-19 centre, a small unit that was set up to cater for COVID-19 patients. At the time of the study (between January 2021 and March 2021 during the second wave of COVID-19), the ward would have about 20–25 patients, with each nurse having a case load of about 6 patients per shift. The total number of nurses assigned for that ward at the time of the study was 12. The doctors (a team of six) who were providing care and examining the COVID-19 patients were not specifically designated for the centre, and they also looked after other medical wards. It is one of the major centres designated for the provision of healthcare services for COVID-19 patients in Zimbabwe. In addition, the centre acts as a referral facility for the management of COVID-19 in the southern region of the country. To recruit the participants, the centre managers distributed information sheets about the study and screening questionnaires to potential study participants on behalf of the researchers. The inclusion criteria for this study were healthcare workers (nurses and doctors) who provided care to COVID-19 patients. Those who met the inclusion criteria and agreed to participate in the study provided their contact details. The researchers then collected these forms, conducted follow-ups through phone calls and arranged the dates and preferred times for interviews. The sample consisted of 10 participants, the majority (*n* = 6) of whom were nurses, whilst the remainder (*n* = 4) were medical doctors working in the COVID-19 unit at one of the central hospitals in Zimbabwe. The demographic data of the participants are displayed in Table 1.

**TABLE 1:** Demographic data of the study participants.

Participant code	Age range	Gender	Profession	Years of experience
Lis	31–35	Female	Medical doctor	7
Fen	36–40	Female	Nurse	14
Vue	31–35	Male	Medical doctor	8
Thes	31–35	Female	Nurse	6
Noma	31–35	Female	Nurse	5
Zah	25–30	Female	Medical doctor	3
Ndue	31–35	Male	Medical doctor	4
Essy	31–35	Female	Nurse	11
Roe	36–40	Female	Nurse	16
Tee	41–45	Female	Nurse	11

### Ethical considerations

Protecting the rights of the participants is considered a significant ethical issue (Gray, Grove & Sutherland 2017). Therefore, to adhere to the study protocol, informed consent documents and research instruments were reviewed and approved by the University of South Africa’s College of Human Sciences Research Ethics (National Health Research Ethics Committee registration number Rec-240816-052) and the Medical Research Council of Zimbabwe (MRCZ/A/268) before the commencement of the study. Permission to conduct the study was also obtained from the Ministry of Health and Child Care and the centre manager. All the participants gave verbal consent before beginning to participate in the study. As part of the consent process, participants were informed that participation was voluntary and that the interviews would be audio-recorded. Participants were also informed that they were free to decline or discontinue participation at any time if they wished to do so. Participants were assured both verbally and in writing that data would be handled confidentially and that the results would be reported in such a way that identification of the informants would be impossible. All identifiers were removed from the transcripts and pseudonyms were utilised to enhance anonymity, and the data were stored securely on a password-protected computer. The study participants were provided with a detailed information sheet that explained the details of the study, and they gave informed consent after assimilation of essential information.

### Data collection

A pilot study was conducted, involving three nurses who were not part of the study and who worked in another department in the hospital. This process assisted the researchers in refining the interview guide, and this led to minor adjustments. Data collection was guided by an interview guide. The development of the interview guide was based on a literature review, and the aim of the study guided the questions. The interview guide questionnaire consisted of semistructured, open-ended questions, with follow-up questions for further clarification.

Because of the potential risk associated with the spread of COVID-19, all the interviews were conducted virtually through a cell phone call that was audio-recorded. An interview guide guided the interview process (see Appendix 1). Before the interview, the researchers informed the participants that the questions would focus on their experiences in providing care to COVID-19 patients since the onset of the pandemic. Individual virtual interviews were conducted by the first author. The interviews lasted 60 min or more. The sample size of 10 participants was determined by saturation, which refers to the point at which the data collection process fails to yield new information relevant to the study (Korstjens & Moser 2018). Data saturation was attained (at eight participants) when no new information emerged and there was only repetition of previously collected data. Two more additional participants were interviewed before closure was reached at 10 participants.

### Data analysis

All audio-recorded interview data were transcribed verbatim into written text. Two researchers analysed the transcripts independently using an IPA framework (Smith & Osborn 2015). A third person, on the other hand, acted as an independent co-coder and conducted the open coding of each transcript. Each researcher read each transcript several times and listened to the audio-recording a few times. The following steps, as outlined by Smith and Osborn (2015), were followed: (1) reading and rereading the transcript; (2) note-taking and developing emergent themes; (3) clustering the emergent themes; (4) crafting a master table of themes – composed of superordinate themes, subthemes and extracts from the interviews; (5) examining and comparing the similarities between the master tables of the themes; and (6) compiling a single master list – composed of a superordinate theme, themes and subthemes. Following this process, the research team then met to compare and discuss their respective master table of themes. The team had a consensus discussion and agreed on the final master table, composed of superordinate themes, subthemes and associated excerpts from the transcripts (Table 2) (Creswell & Creswell 2018; Smith & Osborn 2015).

**TABLE 2:** An example of the data analysis process.

Meaning units	Codes	Subtheme	Theme	Superordinate theme
‘At times, we go on without overshoes. The limitation is in quantities of PPE provided; we have to literally beg to get PPE.’	Indications of shortage of PPE	Inadequate PPE	Resource limitations	Service provision in the context of COVID-19
‘If I see a COVID-19 positive patient when I am moving on to see the next patient, it is not possible to change PPE. We use the same gown and mask the whole day. We would want to change, but PPE is inadequate.’				

PPE, personal protective equipment; COVID-19, coronavirus disease 2019.

## Results

The results section presents the healthcare workers’ experiences of providing care to COVID-19 patients. Following data analysis, the following superordinate themes emerged: human resource-related challenges, limited material resources and impact on the wellness of healthcare workers. Each superordinate theme has several themes and subthemes as presented in Table 3.

**TABLE 3:** Provision of care in the context of coronavirus disease 2019.

Superordinate themes	Themes	Subthemes
Human resource related challenges	Staff preparedness	Suboptimal staff preparation
		Adequate staff preparation
	Support system	Lack of institutional support
		Support from colleagues and peers
		Support from the family
Limited material resources	Resource availability and adequacy	Inadequate personal protective equipment
		Limited equipment and medical supplies
Impact on the well-being of healthcare providers	Stigmatisation and discrimination	Alienation by family members
		Discriminatory attitude of the community members
	Psychological effects	Fear and anxiety
		Stress

## Human resource-related challenges

This superordinate theme focuses on the human resource-related challenges that were experienced by healthcare workers as they provided care to COVID-19 patients. It is composed of two themes, namely staff preparedness and support system.

### Staff preparedness

#### Suboptimal staff preparation

It emerged that staff preparation was suboptimal for the healthcare workers before they started providing care to COVID-19 patients (a novel disease).

‘I feel that I was not adequately prepared or supported before I started working at the COVID-19 centre. I was scared by the way people were dying. I had not even attended the COVID-19 workshops. I was scared because I did not know where to start.’ (Fen, nurse, 14 years professional experience)

‘I was just appointed [or] assigned to go and work at the COVID-19 centre without being prepared or given an option. I was never counselled about the proposed move. I just saw it on the change list. They did not even ask me if I am willing to change or not, even to understand my social dynamics. I was never happy or understood the criteria used for assigning [health care] providers to work at the centre.’ (Essy, nurse, 11 years professional experience)

#### Adequate staff preparation

Whilst some participants felt they were not ready or prepared to work at a COVID-19 centre, it was the opposite for others, who thought they were adequately prepared and/or that it was important to sacrifice and provide care for the benefit of patients. The few who felt that they were somehow prepared had come across COVID-19 patients in the medical wards before the COVID-19 centre was opened. In addition, a few had been exposed to a form of sensitisation on COVID-19. This is reflected in the following extract:

‘I was informed that I was going to work at the COVID centre. It was not hard for me because I worked in a medical ward before and came across COVID-19 patients. Because of my previous experience, I feel I was prepared for this role. Doctors from the infectious disease control department told us that we would be safe as long as we used PPE correctly. I also felt, as a nurse, it would be good to assist people.’ (Thes, nurse, 6 years professional experience)

‘For me, it was not something new because I had looked after COVID patients before this centre was opened. Also, the fact that these were human beings, and I felt I could provide the care for the benefit of the patients.’ (Noma, nurse, 5 years professional experience)

### Support system

This theme describes the availability or absence of a support system for frontline healthcare workers before the commencement of healthcare provision at the COVID-19 centre and during their execution of duties. The experiences on support are outlined under lack of institutional support, support from colleagues and peers and support from the family subthemes.

#### Lack of institutional support

Participants generally felt that there was a lack of support from the Ministry of Health. It was established that the institution does not have a system of supporting or checking how the frontline healthcare workers who provided care to COVID-19 patients were coping. This is illustrated in the excerpt below:

‘There is no support system. No one really asks how we are coping as we provide services. If you feel like crying after losing a client you thought would make it, you just cry on your own. Feedback or review meetings are currently not available.’ (Lis, medical doctor, 7 years professional experience)

‘Anyone with symptoms or who gets sick finds their way. If the main casualty does not have rapid antigen tests, there is no way of testing except to go to private laboratories at your own expense.’ (Roe, nurse, 16 years professional experience)

Because of the nature of the virus, the staff working with COVID-19 patients cannot eat after changing into personal protective equipment (PPE). Alternative ways had to be devised to address staff nutrition. In this regard, one participant noted:

‘They suggested that we have meals at home. This is because one cannot have meals while putting on PPE. Some do not feel like eating in that department. But the hospital had said they would provide breakfast, lunch and supper. However, at the moment, they provide bread, you have to bring your juice. We only have our meal when we knock off.’ (Fen, nurse, 14 years professional experience)

Billings et al. (2021) call for a differentiated approach to providing support to healthcare workers during COVID-19. The authors also indicate that for the support to be effective, it has to be coherent, consistently communicated and readily accessible, yet in the context of this study, institutional support was not available. In addition, Greenberg and Tracy (2020) advocate for hospital managers to be trained in having ‘psychologically savvy conversations’ in order to create awareness on mental issues and enhance their mental well-being.

#### Support from colleagues and peers

However, the following extract shows that the health workers often showed concern for each other and maintained solidarity with their coworkers:

‘The hospital does not have a support system. We wanted to set up one, but it has not materialised. So more support comes from friends. Those that are religious resort to spiritual support. Worse still, there is a lockdown. Some places to which many people can go are closed. Sometimes we resort to getting alcohol in order to sleep.’ (Vue, medical doctor, 8 years professional experience)

‘We share experiences with colleagues through a WhatsApp group. However, it is not enough, it would be good if debriefing or sharing of experiences or fears or anxieties was done in a professional environment, for example, through a psychologist or an organised system.’ (Essy, nurse, 11 years professional experience)

In this study, peer support has proven to be an effective support system for healthcare workers, particularly during trying times like COVID-19 (Suresh, Alam & Karkossa 2021). Similarly, Gidugu et al. (2015) indicate that individual peer support facilitates provision of beneficial interactions and health resources during challenging situations such as COVID-19.

#### Support from family

Whilst the reactions of family members differed, some participants reported that their relatives were supportive, as noted below:

‘My family stays in another town. They were very concerned about my working at a COVID-19 centre and expressed this fear of the COVID-19. However, they were a pillar of my strength and very supportive. They would phone every day to check on me.’ (Ndue, medical doctor, 4 years professional experience)

‘My family and relatives know that I work at a COVID centre, and they have been very supportive.’ (Fen, nurse, 14 years professional experience)

Klop et al. (2021) posit that family support is crucial for healthcare providers on the frontline, especially during trying times like the extreme COVID-19 situation.

## Limited material resources

This superordinate theme is about the experiences of frontline healthcare workers regarding the provision of care to COVID-19 patients in a healthcare setting with limited material resources. Resource availability and adequacy is the theme that emerged.

### Resource availability and adequacy

This theme is about the availability and adequacy of equipment and medical supplies that were meant to enhance provision of care for COVD-19 patients. The healthcare providers reported challenges associated with limited resources. Two subthemes emerged: inadequate protective equipment and limited equipment and medical supplies.

#### Inadequate personal protective equipment

The frontline healthcare workers felt the institution was under-resourced. Inadequacy of PPE was cited often. One participant had this to say:

‘We get overshoes, headgear, a face shield, N95, 2 surgical masks. At times, we go on without overshoes. The limitation is in [*the*] quantities of PPE provided; [*you*] have to beg to get PPE, literally.’ (Lis, medical doctor, 7 years professional experience)

‘The PPE supplies are not enough for the people that work at the centre. Therefore, to try and manage the situation, we have fewer people putting on PPE and going into the ward. Other nurses would remain outside the unit. This creates a lot of strain and burnout on the providers that would be providing care for the COVID-19 patients.’ (Fen, nurse, 14 years professional experience)

The increased demand for PPE and its subsequent shortage in healthcare settings have been noted in other studies (Alhalaseh et al. 2021). The COVID-19 pandemic has adversely affected the supply chain of PPE at a time when the commodity is in high demand (Cai et al. 2020).

#### Limited equipment and medical supplies

In addition, there were challenges related to limited equipment and medical supplies, resulting in out-of-pocket expenses for patients, as one participant noted:

‘Most of our patients are of low-income status, yet they are required to purchase some medicines that are not available from the hospital pharmacy. Some clients have succumbed to some illness before they could have bought the medication. Oxygen is available, but there were episodes where it was on and off.’ (Zah, medical doctor, 3 years of experience)

In some instances, COVID-19 patients with comorbidities had to obtain their own medicines and/or related equipment, as observed in the extract below:

‘Sometimes medicines, such as ceftriaxone and azithromycin, are not available at the hospital and patients would be required to buy [them], yet they cannot afford to. The equipment for managing chronic conditions is inadequate; for example, patients are requested to buy glucometers, particularly if there are no Glucostix and the patient is a newly diagnosed diabetic.’ (Ndue, medical doctor, 4 years of experience)

Whilst in Zimbabwe the shortage of medicines could be attributed to an ailing healthcare system, the situation has been further exacerbated by the COVID-19 pandemic, as other studies have demonstrated (Mukwenha et al. 2020). These scholars argue that the closure of borders have affected the stockpile of medicines which are imported. Ying et al. (2021) attribute the shortage of medicines to COVID-19-induced interruptions to the supply chain.

## Impact on the well-being of healthcare providers

This superordinate theme demonstrates the impact of COVID-19 on the well-being of healthcare providers who work at the COVID-19 centre. Two themes that emerged include stigmatisation and discrimination as well as psychological effects.

### Stigma and discrimination

#### Alienation by family members

Because of the high chance of viral transmission, health workers, who are on the frontline in combating COVID-19, are often shunned and perceived to be virus carriers. The following extracts exemplify the stigma and discrimination some underwent:

‘Initially, my family felt I needed to stay at the hospital for the duration of my work at the COVID-19 centre. Alternatively, the general feeling was that I was supposed to resign from work and start all over again after COVID-19. Family members were generally uncomfortable in my presence.’ (Essy, nurse, 11 years professional experience)

‘My mother stays in the neighbourhood. She told me that I should not bother to visit them but stay at my place and rest. I could see she was scared of me. As a result, I would phone them to come and collect the groceries from the bus stop where I would have dropped them.’ (Lis, medical doctor, 7 years professional experience)

‘I have experienced stigma and discrimination. I have a brother who would say, “I have a sore throat; I think this is your COVID-19 you brought at home.” The feeling is that any COVID-19 at home has been brought by me, who works at a hospital.’ (Thes, nurse, 6 years professional experience)

#### Discriminatory attitudes of the community members

The frontline health workers experienced some form of stigma and discrimination, particularly those who used public transport. The following extracts illustrate their experiences:

‘I was using public transport. I could not get lifts as before. Punctuality for duty became difficult. In public vehicles such as taxis, people avoided me. Word spread that I could be carrying COVID from the hospital.’ (Roe, nurse, 16 years professional experience)

‘I got a lift wearing my nurse’s uniform. Then I coughed in the car and was requested to drop off before getting to my destination. I felt traumatised and stigmatised, considering that I sacrifice my life to provide care to patients.’ (Tee, nurse, 11 years professional experience)

Instances of stigma and discrimination are not unique to this study setting, as studies by Bagcchi (2020) and Taylor (2020) demonstrated how during the pandemic healthcare workers were often shunned, ostracised and discriminated against.

### Psychological effect

#### Fear and anxiety

Often, participants found themselves overwhelmed by anxiety and fear. The psychological impact of work-related stress was huge, as captured by the following excerpt:

‘The first few weeks of December were very hectic, and many patients were dying within a few hours of admission. We were seeing high rates of mortality, so it was emotionally draining, and I had a lot of fear and anxiety. I wished there was provision for diversional therapy or psychological support in this kind of stressful situation.’ (Noma, nurse, 5 years professional experience)

‘I was afraid for my family because of the disease. I was afraid of going home. I also had fears associated with dying, since COVID-19 was taking a lot of lives. There are no protocols on what happens when a member of staff contracts COVID-19. When one falls sick, it is your responsibility and that of the that of the family. This has been my major worry.’ (Ndue, medical doctor, 4 years professional experience)

#### Stress

It also emerged that the frontline healthcare workers who work at the COVID-19 centre felt stressed and traumatised by the experience of having to care for colleagues and losing many patients to COVID-19, as reflected by the extracts below:

‘[*N*]ursing other healthcare workers as COVID-19 patients is a shaky situation; it also affects us as providers; it affected me emotionally. I was traumatised and imagined myself in a similar situation.’ (Zah, medical doctor, 3 years professional experience)

‘The job is emotionally draining and stressful. You see a patient and you think he is recovering; the next moment, the patient has died. When you see [*a*] patient and put all to it, in terms of management, you would want to see them discharged home, but it does not happen that way. We have seen a lot of clients dying.’ (Tee, nurse, 11 years professional experience)

Frontline healthcare workers felt stressed as a result of the inadequacy of a transport system to travel to and from work, as shown in the following extract:

‘I felt frustrated and stressed because of this transport issue, having to go through the roadblocks. … The Ministry should have organised transport for health workers, particularly during the pandemic. I would spend the whole day providing care for patients, and when going home, people would not even want me near them in buses.’ (Roe, nurse, 16 years professional experience)

These findings on the psychological effects of COVID-19 on the mental well-being of healthcare workers are consistent with the findings of studies elsewhere. A study in China by Lam et al. (2020) established that healthcare workers who perceived themselves to be at risk of contracting COVID-19 experienced anxiety and symptoms of depression. According to Teo et al. (2021), in response to the COVID-19 lockdown measures and related adaptations in the work environment, healthcare workers experienced stress, anxiety and burnout during COVID-19. Related studies in Italy (Naldi et al. 2021) and in China (Chen et al. 2021) found that stress and anxiety have a bearing on the mental health outcomes of healthcare workers during the COVID-19 pandemic.

## Discussion of findings

The aim of this study was to explore and describe the experiences of healthcare workers who provided care to COVID-19 patients and the effect the novel disease has on healthcare service delivery. Our findings show that the COVID-19 pandemic had a negative impact on the healthcare system, resulting in frontline healthcare workers being overwhelmed as a result of the immense physiological and psychological pressures they have been experiencing. The discussion will focus on the following key findings and their implications: human resource-related challenges, limited material resources and the impact on the well-being of healthcare providers.

Except for those healthcare workers who had prior experience of caring for COVID-19 patients (before the opening of the COVID-19 centre), the staff were barely prepared for the epidemic. The study demonstrated that there was suboptimal staff preparation of healthcare workers prior to commencing COVID-19 activities. Similarly, Al-Ashwal et al. (2020) in a study in Yemen found that healthcare providers were inadequately prepared to provide care in a COVID-19 setting. In hindsight, adequate staff preparation was necessary, as the epidemic disrupted the pace of life and introduced the unfamiliar and uncontrollable. The COVID-19 outbreak highlighted the fragile nature of the Ministry of Health’s psychological resilience and also amplified the need for attention to be paid to the psychological state of health workers (Elkholy et al. 2020). This author has advocated for proper mental health support for healthcare workers to ensure continuity of healthcare services during such epidemics. In the absence of an agency that coordinates and plans psychological interventions for the population, the involvement of mental health professionals in the COVID-19 task force is necessary. These mental health professionals would advise the government on their psychological or mental health policies. This fact is bolstered by Highfield (2020) and Cabarkapa et al. (2020), who argue that organisational leaders need to understand the needs of their workforce. During the preparatory stage, it is important to identify the most vulnerable team members to put plans in place to support them. In the context of this study, the frontline healthcare workers, who had had no prior experience with COVID-19, should have been consulted and psychologically prepared before deployment to the COVID-19 centre.

In the context of this study, healthcare workers expressed fears and concerns about transmitting the virus to their loved ones, who in some cases shared the same fears. In addition, frontline healthcare workers experienced exhaustion, the pain of losing some patients and the anxieties of having to care for coworkers, in addition to their concerns about the risk of infection. Studies by Liu et al. (2020) underscore the importance of safeguarding the morale and mental health of healthcare workers, as it can impact on the success and outcome of the delivery of health services. Evidence also highlights that during COVID-19, the provision of quality care depends on the mental well-being of the healthcare service providers (Chersich et al. 2020; Cullen et al. 2020).

These findings are similar to those of Adams and Walls (2020) and Liu et al. (2020), where frontline healthcare providers who lived at home had concerns about transmitting the virus to family members. To address this challenge and reduce the levels of anxiety experienced by healthcare workers, the authors of this study would recommend supportive conversations with healthcare providers such as separation of living spaces, changing clothing and immediately showering when reaching home after duty. Additionally, the authors recommend supportive counselling to address the fears that the family members and/or significant others may have. This is in keeping with the recommendations by Cabarkapa et al. (2020), who calls for psychosocial support tailor-made to suit the specific needs of the healthcare workers to empower them as they combat the epidemic. Related to this, Okediran et al. (2020) also advocate for a mental health assessment of the healthcare worker and the provision of support by psychologists during this time that healthcare workers are caught up in this campaign to provide care for COVID-19 patients. Hyun et al. (2020) found that a team of multidisciplinary experts in mental health using different media, including virtual consultations through telephonic video chats, can effectively support patients. A similar strategy of virtual support can go a long way in supporting the mental well-being of frontline healthcare workers who care for COVID-19 patients. The mental health and well-being of frontline healthcare workers are important, as is emphasised by Tomlin, Dalgleish-Warburton and Lamph (2020), who developed a model to provide psychological support for frontline healthcare workers in the context of the COVID-19 environment.

This study found inadequate or lack of support systems for the healthcare workers. Participants felt the institution could have done more to help them cope with their fears and anxieties, especially after losing many patients. If healthcare workers are not given social support, their anxiety levels rise, and the quality of their sleep is affected. This was the finding in China, by Xiao et al. (2020). A study by Williams et al. (2020) found that different governments provided different types of support to boost the morale of health workers. According to another author, Poland, Malta and Romania provided free accommodation for their staff, who were isolated from their families, whilst Hungary provided free access to public transport. This study found no such support for frontline healthcare workers who worked at the COVID-19 centre in Bulawayo, who often had challenges accessing public transport in the midst of COVID-19 restrictive measures. A study in Singapore, by Chan and Huak (2004), on SARS amongst healthcare workers found that support from coworkers, coupled with clear communication on precautionary measures and directives, can reduce psychiatric symptoms. If the staff are confident about the infection control measures that are in place, it mitigates and facilitates their adaptation to stress. According to the participants, the support was mainly provided by coworkers and immediate family members. Another recommendation to support frontline healthcare workers would be to use a social media intervention such as the Vula platform in South Africa. This intervention has been effective in offering mental health support for healthcare workers and has been demonstrated to be effective in reducing stress (Hu et al. 2020).

Participants indicated that the institution had resource constraints, namely PPE, medication and other related equipment for managing COVID-19 patients. The World Health Organization, in 2020, issued guidelines for the protection of both the healthcare workers and the public (WHO 2020a). Amongst other things, the guidelines call on governments to ensure the availability of PPE in adequate quantities and the appropriate fit. In addition, governments are called upon to ensure adequate training on the use of PPE. Contrary to these guidelines, the study found that the provision of PPE was inadequate. This led to a limited number of staff being issued with PPE to be able to get into the COVID-19 wards, resulting in burnout and overwork. In addition, the training of staff on the appropriate use of PPE was also inadequate. A study by Eftekhar Ardebili et al. (2021) had similar findings: a shortage of protective devices and overwhelming workload. Cai et al. (2020) and Ghinai et al. (2020) assert that the provision and proper use of PPE can go a long way in mitigating the risks associated with healthcare workers’ infection. Therefore, it is recommended that institutions provide adequate PPE and train the users on the appropriate use of such gear. The inadequacy of PPE provisions for frontline healthcare workers found in this study is worrisome, as evidence from Italy and the United States (US) suggests that inadequacy in PPE contributes to higher infection and mortality rates (Balmer & Pollina 2020; Jacobs, Richtel & Baker 2020).

Apart from the inadequate provision of PPE, the study found that the institution also suffered from a shortage of medicines (antibiotics and medicines for noncommunicable diseases such as diabetes and hypertension), often forcing COVID-19 patients to purchase them at their own expense. This impacted negatively on low-income patients. Resource constraints are not unique to this study. Studies in Nigeria by Okediran et al. (2020) and in Cameroon by Ojong (2020) had similar findings. According to the WHO (2020c), out-of-pocket expenses for patients are prevalent in Africa, particularly in Cameroon, Comoros, Equatorial Guinea, Guinea Bissau and Nigeria, but has been exacerbated by the COVID-19 pandemic.

This study also found that frontline healthcare workers experienced social ostracism, both at family and community levels. Similarly, other studies showed that healthcare workers were being alienated by their families because of employment related to COVID-19 (Chaudhary et al. 2020; Dang et al. 2020). Participants in the present study faced challenges because spouses or family members felt they were a risk to the family. As a result, they were encouraged to either stay at the hospital or resign from the job. Given such attitudes and the nonsupportive environment, the management of COVID-19 becomes difficult. At the community level, healthcare workers experienced discriminatory attitudes from the community and faced challenges accessing public transport or lifts from neighbours. The results of this study are in line with the study findings by Taylor et al. (2020), who found that the public was afraid of COVID-19 and avoided healthcare workers. Studies elsewhere identified stigma and discrimination against frontline healthcare workers during the COVID-19 pandemic (Juane et al. 2020; Park et al. 2018). A study by Mostafa et al. (2020) showed that many Egyptian healthcare workers encountered stigmatisation from neighbours. Studies by Withnall (2020), *The Economist* (2020) and the WHO (2020a) found that there were attacks on healthcare providers in countries such as India, the USA and Australia, where frontline healthcare workers were being beaten, threatened and evicted from their homes. Singh and Subedi (2020) call for accurate information dissemination to dispel myths and to create an enabling environment to combat COVID-19.

### Limitations of the study

The study has some limitations which need to be acknowledged. The study was conducted during the early phase of the pandemic. The situation might have changed. It was limited to healthcare providers in Bulawayo; therefore, the findings report only the experiences from this province. However, the same study may be replicated in other cities or countries in the region.

### Implications of the study

This study provides early evidence on the challenges faced by healthcare workers as they provided care to COVID-19 patients. The challenges identified from this study will inform policymakers, programme planners and health facility managers involved in the response to COVID-19 or any future pandemic. The provision of differentiated comprehensive psychosocial support is critical to enhance the mental well-being of healthcare providers. Many challenges emerged from this study that call for further investigation and research.

## Conclusion

The study found that frontline healthcare workers needed to have been trained in disease management before the country had to deal with the disease outbreak and its intensification. The World Health Organization had developed guidelines and protocols for the management of COVID-19 patients, but some healthcare providers in this study had not been sensitised about these. Health institutions must provide all-around psychological support for all healthcare workers in a formal structured manner. The Ministry of Health and Child Care must ensure that healthcare workers have adequate equipment to manage COVID-19 patients. Additionally, the frontline healthcare workers need support in adequate PPE, reasonable work schedules, monitoring, supervision of implementation and adherence to infection control protocols. The COVID-19 pandemic brings with it unique and challenging experiences for frontline healthcare workers. The intensive work has drained healthcare providers physically and emotionally, and this calls for the provision of comprehensive support and increased access to individualised mental health services. Whilst the study was confined to one centre, the findings are significant in that they may be used in the furtherance of healthcare service provision in the context of COVID-19.

## Appendix 1: Interview guide for frontline health care providers

### Brief biographical information

Age: ………………………………………

Gender: ……………………………………

Years of experience: ………………………‥

Professional Background: …………………….

### Grand tour Question

Describe your experiences of providing care to suspected or confirmed cases of COVID-19.

### Probing Questions

- How did you feel about caring for a suspected/confirmed patient with COVID-19?
- How did you cope with your fears/anxieties?
- What challenges did you encounter?
- What suggestions would you recommend for future care?
- Any suggestions on how health care workers infected or affected by COVID-19 could be supported?
